# Haptic Search for Hard and Soft Spheres

**DOI:** 10.1371/journal.pone.0045298

**Published:** 2012-10-08

**Authors:** Vonne van Polanen, Wouter M. Bergmann Tiest, Astrid M. L. Kappers

**Affiliations:** Helmholtz Institute, Utrecht University, Utrecht, The Netherlands; McMaster University, Canada

## Abstract

In this study the saliency of hardness and softness were investigated in an active haptic search task. Two experiments were performed to explore these properties in different contexts. In Experiment 1, blindfolded participants had to grasp a bundle of spheres and determine the presence of a hard target among soft distractors or vice versa. If the difference in compliance between target and distractors was small, reaction times increased with the number of items for both features; a serial strategy was found to be used. When the difference in compliance was large, the reaction times were independent of the number of items, indicating a parallel strategy. In Experiment 2, blindfolded participants pressed their hand on a display filled with hard and soft items. In the search for a soft target, increasing reaction times with the number of items were found, but the location of target and distractors appeared to have a large influence on the search difficulty. In the search for a hard target, reaction times did not depend on the number of items. In sum, this showed that both hardness and softness are salient features.

## Introduction

In daily life, we encounter many compliant objects. Common examples of soft objects are the sponge one uses for washing up and the stuffed animals children play with. It is important to be able to distinguish efficiently between soft and hard objects, for instance when judging the ripeness of fruit. Also in medical palpation procedures sensitivity for compliance is necessary, since an increased softness or hardness of a body part (e.g. the skin) can indicate a disease.

Several studies have been performed to determine the discrimination threshold (just noticeable difference, JND) in softness or hardness perception [1]–[4]. The ratio of the JND and the intensity of the stimulus is called the Weber fraction, which gives an indication of discrimination performance. In the literature, Weber fractions of compliance range from 13–25%.

The discrimination threshold indicates how well one can distinguish between two stimuli that differ in compliance. Other interesting questions concern the efficiency with which hardness or softness is perceived and whether these features are salient. Salient features are easily accessible object properties that are almost instantly perceived. Therefore, these properties are likely to be important for the recognition of objects and used in the early phases of object recognition [5]. It must be noted, though, that next to these bottom-up theories, other models do not place a large role on these properties, but propose a more top-down guidance of attention [6]. In vision research, many salient features are stated to be visual primitives. These primitives are defined as features that lie at the basis of visual perception and are automatically picked up, without the need of focused attention [7]. Possibly the same principles hold for haptics, and investigating saliency might thus teach us something about the basic properties of haptic perception.

The saliency of an object feature can be investigated in a search task, where one has to determine whether a target is present or not among a variable number of distractors. If a target object property is easy to find, it stands out among the distractor objects' properties. This is called the pop-out effect, which has originally been described in visual search [7], [8]. For example, if the letter L is shown among a number of plusses (+), one does not need to look at each letter to determine whether it is an L or not; the L is spotted instantaneously. On the other hand, if the L had been placed between Ts, the target and distractor are more difficult to distinguish and the target does not pop out among the distractors [9].

If the target is easily distinguished from the distractors, a parallel search strategy can be used, in which all items are examined at once. As a result, the time to search for the presence of a target can be very short and is independent of the number of items. If the target is more difficult to distinguish from the distractors, the items have to be explored one by one. This is called a serial strategy and is less efficient. In a serial strategy, the time to search for the presence of a target increases if more items need to be searched. Following this reasoning, the efficiency of search can be measured by plotting the reaction time, i.e. the time to decide whether or not a target is present, against the number of items that are explored. The slope of the regression line fitted through the reaction time data is called the search slope. The search slope indicates the difficulty of the search and the search strategy: a flat slope indicates a parallel strategy, whereas a positive slope implies a serial strategy. Therefore, the search slope can be used as a tool to measure search efficiency and the saliency of the target feature. However, caution must always be taken with the interpretation of slope values, because the exact distinction between the two search strategies is not very strict. In the visual literature, a range of search slopes can be found [10]. Therefore, the slopes must be interpreted in the context of the task. For instance, two slopes can differ significantly if a search asymmetry occurs. In a search asymmetry, a target property is easy to find among a certain distractor property, whereas this is not the case the other way round. For example, a rough item pops out among smooth items, whereas a smooth item is difficult to find among rough items [11]. This search asymmetry indicates that roughness is a more salient feature than smoothness.

Haptically searching for objects can be very efficient. Research into haptic search reveals a number of haptic salient features (e.g. roughness [11], temperature [12], edges and vertices [13], movability [14] and hole vs. no hole [5]). In this study, we wanted to investigate the saliency of hardness and softness. Lederman and Klatzky [5], [15] have investigated haptic search for various properties and found very efficient searches and low search slopes in the search for a hard item among soft items and also for a soft item among hard items. This would imply that both features are salient and no search asymmetry is present. They provide basic evidence of the saliency of compliance, but it remains unclear whether these results also apply to other contexts. In the study of Lederman and Klatzky [5], a static position was used, where stimuli were pressed against the fingertips of participants. This set-up limited the exploration by only making small finger movements possible and induces a passive role for participants. We wanted to use a more natural, active approach by letting the participants grasp a bundle of objects with the hand. A second advantage of this set-up is that it requires a different exploratory movement than used in the Lederman and Klatzky study [5]. In another paper, they have described the optimal exploratory procedure for the perception of compliance as “pressure” [16]. An example of this is pressing an object that lies on the table, but in daily life one often squeezes an object between the fingers to determine its hardness or softness. By grasping a bundle of objects, several exploration strategies can be used and objects can be manipulated in the hand.

To sum up, in this study we wanted to further explore the saliency of hardness and softness in a haptic search task that involves active grasping of multiple objects. In this way, exploratory movements are not restricted and perception is not limited to a small part of the hand; the whole hand can be used to determine compliance, which might be more efficient when multiple objects are explored. The main question is whether a pop-out effect can be found for hardness or softness or both in an active search task.

Two experiments were performed in this study, in which participants had to determine whether a target was present among distractors. In Experiment 1, participants had to grasp a bundle of hard and soft items. Two sub-experiments were performed, in which the difference in compliance between target and distractor varied. In experiment 1a the difference in compliance was small, whereas in experiment 1b the difference was large. We hypothesized a more efficient search with a larger difference in compliance. In Experiment 2, the items were placed on a display and participants had to press their hand on the soft and hard objects. In this last experiment, manipulation of the separate objects is not possible, but since the participants are free to press their hand or fingers on the display in a way they prefer, the task is still active and not limited to perception at the fingertips. Furthermore, because the objects are placed on a display, weight cues cannot influence the task.

## Experiment 1

### Methods

#### Participants and ethics

Ten participants (7 males) with a mean age of 21±3 years were recruited for the experiment. All were strongly right-handed as confirmed by Coren's test [17], which is a simple test with questions about hand-use in different situations. Only right-handed participants were chosen for practical purposes, since the experimental set-up was designed for right-hand use. They used their dominant hand for performing the experiment, because we were interested in the best possible performance. Participants gave their written informed consent prior to participation and were paid for their contribution. This study was conducted in accordance with principles as stated in the declaration of Helsinki. Participants performed tasks that did not deviate from daily life. Therefore, the “Medisch Ethische Toetsingscommissie” (Medical ethical review committee) of Utrecht University declared that ethical approval was not necessary.

#### Apparatus

**Figure 1:**
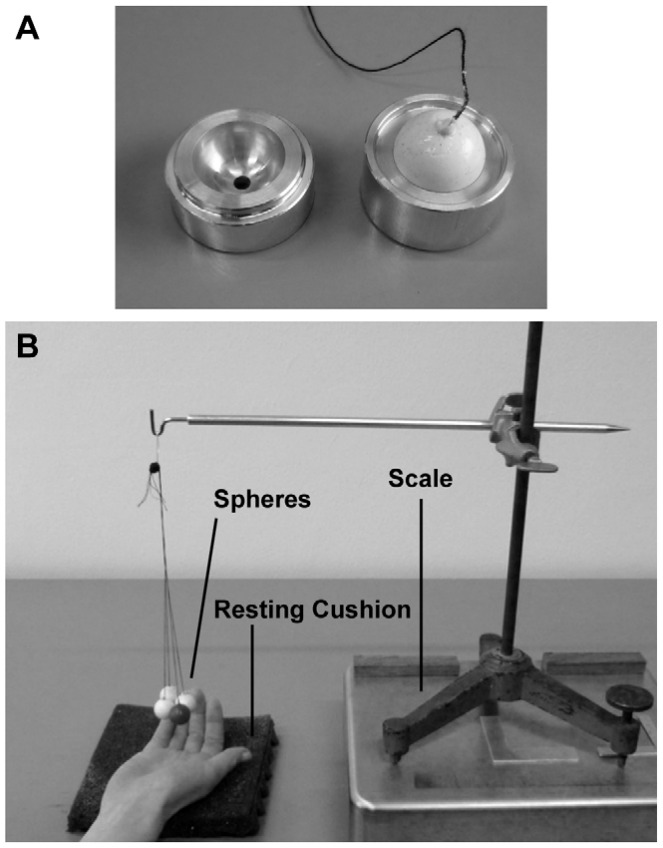
Experimental set-up for **Experiment 1** A: The two halves of the mould, with a sphere in the right half. B: The experimental set-up of Experiment 1. A bundle of four spheres (one dark target sphere) hangs from a tripod placed on a weighing scale. Underneath the hand lies a resting cushion.

The stimuli consisted of spheres with a radius of 9.3 mm, which was the same size as used in the study of Plaisier et al. [13] that used a similar search task. Spheres instead of, for example, cubes were used to avoid information about the edges, which are expected to be more salient with harder objects [13]. The spheres were made of silicon rubber (Wacker Silicones) and could be “hard”, “middle-soft” or “soft”. The spheres had an average weight of 4.8 g, 4.0 g and 0.71 g, respectively. The hard and middle-soft spheres were produced by pouring liquid rubber (hard: type M4470, middle-soft: M4500) into an aluminium mould (see Fig. 1A). The spheres were then solidified by the addition of a catalyst (hard: type T40, middle-soft: T12). The soft spheres were hollow. This was realised by pouring circa 0.5 ml (type M4500, catalyst T12) into the mould and turning the mould every 15 or 30 minutes to spread the rubber along the inner sides of the mould while the rubber solidified.

A piece of string was attached to each sphere and the spheres were grouped in bundles of 3, 4, 5, 6 or 7 spheres. Seven spheres was the maximum number of items that could fit comfortably in the hand. A bundle could be hung onto a hook, which was attached to a tripod (see Fig. 1B). The spheres hung approximately 9 cm above the tabletop.

To measure the reaction time, the tripod was placed on a weighing scale (Mettler Toledo SPI A6). When the participants touched the spheres, the resulting weight change started the clock. The end of the reaction time was determined by a vocal response, recorded with the microphone of a headset placed on the participants' heads. The reaction time was sampled with a frequency of 100 Hz. The weighing scale had a delay of 90±20 ms, which was added to the raw reaction time data.

#### Compliance measurements

The compliance of the spheres was measured with an Instron 5542 Universal Testing Machine. A sphere was pressed between two flat metal plates with 1 mm/s, and the force and compression were measured (in steps of 0.1 N per sample). The compression was stopped at 10 N for the hard and middle-soft spheres, and at 2 N for the soft spheres. The lower endpoint for the soft spheres was chosen to make sure they remained intact. For each compliance type, 5 spheres were measured 3 times, totalling 45 measurements.

**Figure 2:**
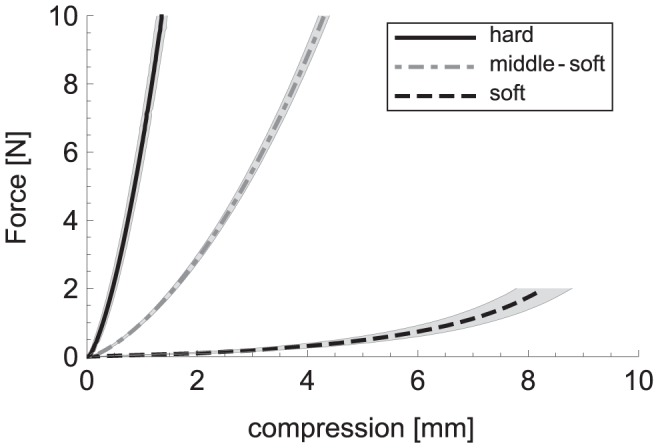
The relation between force and compression for hard, middle-soft and soft spheres. Grey areas represent confidence intervals (2 standard deviations).

The 15 measurements for each compliance type were averaged and the resulting values are displayed in Fig. 2. It can be clearly seen that the three kinds of spheres are very different in compliance. However, it is difficult to determine a compliance value, since the lines are not straight. This is because the surface area that was pressed increased with more compression, i.e. the spheres got flatter. To be able to compare the local compliance values of the three kinds of spheres, regression lines through 5 data points around a reference value of 1.5 N were calculated. The resulting compliance values were 5.8 N/mm, 1.7 N/mm and 0.68 N/mm for the hard, middle-soft and soft spheres, respectively.

#### Task and procedure

In each trial, blindfolded participants had to grasp a bundle of spheres and determine as quickly as possible whether a target was present or not among distractors. The experiment was divided into two sub-experiments. In experiment 1a, the items were the hard and the middle-soft spheres. The target could be a hard sphere among middle-soft distractors (hard-target condition) or a middle-soft sphere among hard distractors (middle-soft-target condition). In experiment 1b the hard and soft spheres were used. In the hard-target condition, the target was a hard sphere among soft spheres and in the soft-target condition it was the other way round.

The order of Experiment 1 and Experiment 2 was counterbalanced between participants. Within Experiment 1, the order of experiments 1a and 1b was counterbalanced as much as possible between participants. The order of the conditions within an experiment (e.g. whether or not an experiment was started with the hard-target condition) was randomized between participants in a way that each experiment started or ended with a hard target equally often. In each of the four conditions, 20 trials per number of items (3, 4, 5, 6 or 7) were performed by each participant, of which half of the trials contained a target. In target-present trials a single target was present among distractors, whereas in target-absent trials, there were only distractor items in the bundle. The number of items and the target-present and target-absent trials were randomized within a condition. The location of the target was not systematically controlled. However, care was taken that the target was located at different positions throughout the experiment. It must be noted, though, that the target could change position once participants grasped the bundle, and that they could manipulate the items in their hand. Each condition was performed on a different day.

Participants were seated in front of the weighing scale. They were told the nature of the task and instructed to try to determine the presence of a target as quickly as possible, but also to make as few mistakes as possible. Before each trial, they put their flat hand, with the palm up, upon a resting cushion underneath the bundle of spheres. They were instructed to lift their hand and initially grasp the whole bundle, but if necessary explore the spheres individually or drop spheres out of their hand. As soon as they knew whether a target was present or not they responded by calling out the Dutch equivalents of ‘yes’ or ‘no’. They received feedback whether their answer was correct. Before the start of a condition, participants performed at least 20 practice trials continuing until they answered correctly 10 times in a row. This was done to get familiar with the task and to find a fast strategy to perform the task. The maximum number of practice trials needed was 27. Trials answered incorrectly were repeated at the end of the session.

#### Analysis

One trial (0.03%) was removed from the analysis because of a measurement error. In addition, outliers in the reaction time data were removed from further analysis. A trial was considered an outlier if it differed more than 4 standard deviations from the mean, when the trial itself was not included in the calculations of the mean and standard deviation.

The mean reaction times were plotted against the number of items for each participant, separately for each condition and for target-present and absent trials. A straight line was fitted through the data points, giving the search slopes and intercepts. Furthermore, for each trial was scored whether participants dropped spheres out of their hand. The percentage of trials in which this behaviour was seen was calculated for each number of items. For experiment 1a with a hard target, the scoring was incorrect for 1 participant and these data were left out of the analysis for this variable in this condition. For all other variables, all 10 participants were included in the analysis.

A 2 (experiment)×2 (target type)×2 (target presence) repeated measures Analysis of Variance (ANOVA) was conducted on the slopes and intercepts. Post-hoc tests were performed by using paired-sample *t*-tests with a Bonferroni correction. Only relevant comparisons were made. The significance value was set at 0.05.

### Results

#### Errors

The percentage of incorrect answers is displayed in Table 1. More errors were made in target-present trials than target-absent trials. This indicates that participants rarely reported a target that was not there, but more often missed one, which is typical in search literature. In experiment 1a, participants made more errors than in experiment 1b. Especially with a large number of items, more incorrect answers were given.

**Table 1:** Percentage of errors for Experiment 1, for each number of items (3, 4, … 7).

experiment	condition		3	4	5	6	7
1a	Hard	present	0	1	3	10	6
		absent	1	0	1	1	0
	Soft	present	2	3	5	10	14
		absent	0	0	0	0	0
1b	Hard	present	2	0	2	3	0
		absent	0	0	0	0	0
	Soft	present	0	1	2	0	6
		absent	0	0	1	0	0

#### Reaction times

**Figure 3:**
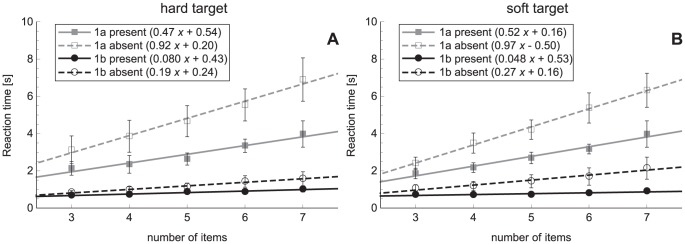
Search slopes for **Experiment 1**. A: Search slopes for experiment 1a and experiment 1b for a hard target. B: Search slopes for a middle-soft target in experiment 1a and a soft target in experiment 1b. Slopes are presented for target-present and target-absent trials separately. Error bars represent standard errors.

Search slopes of experiments 1a and 1b are shown in Fig. 3. Slopes and intercepts can also be found in Table 2. All slopes in Experiment 1 were significantly different from zero. It can be seen that the slopes in experiment 1a are much steeper than the slopes of experiment 1b. An ANOVA on the slopes demonstrated significant effects of experiment (*F*_1,9_ = 52.7, *p*<0.001), target presence (*F*_1,9_ = 19.7, *p* = 0.002) and an interaction between experiment × target presence (*F*_1,9_ = 20.4, *p* = 0.001). Post-hoc tests indicated that there were higher slope values in experiment 1a than in experiment 1b (target present: *p* = 0.003, target absent: *p*<0.001). In target-absent trials, the slope was steeper compared to target-present trials, but this difference was only significant in experiment 1a (*p* = 0.001). There were no significant effects found in the ANOVA on the intercepts.

**Table 2:** Search slopes and intercepts for Experiment 1 and Experiment 2.

		Slope (s/item)		Intercept (s)	
experiment	condition	present	Absent	present	absent
1a	hard	0.47**	0.92**	0.54	0.20
	middle-soft	0.52**	0.97**	0.16	−0.50
1b	hard	0.080**	0.19**	0.43**	0.24
	soft	0.048*	0.27**	0.53**	0.16
2	hard	0.010	−0.013	0.54**	0.91**
	soft	0.12*	0.13	0.43*	1.47

* *p*<0.05,

** *p*<0.01.

#### Search behaviour

**Figure 4:**
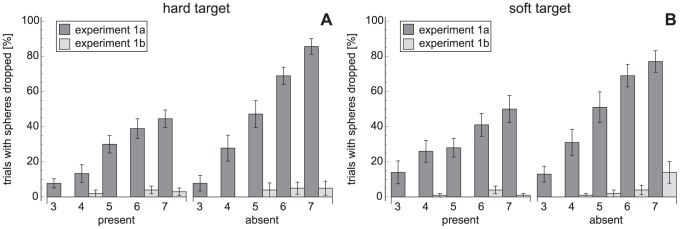
The proportion of trials in which spheres were dropped. A: The hard-target conditions in experiment 1a and experiment 1b. B: The middle-soft-target condition in experiment 1a and the soft-target condition in experiment 1b. Error bars represent standard errors. The bars for experiment 1a with a hard target include 9 participants, the other bars 10 participants.

The number of times a sphere was released from the hand is plotted in Fig. 4. It is clear that many more items were dropped in experiment 1a, especially in the target-absent trials. With more items in the hand, more often an item was released. Typically, the items were released one by one.

## Experiment 2

Another natural way to determine the compliance of an object is pressing it [16]. As mentioned in the introduction, this is still an active search task, because participants are free to actively explore the display. Therefore, a second experiment was performed in which participants pressed their hand upon a display filled with spheres. In addition, this experiment controlled for the possible influence of weight cues in Experiment 1. Since the soft and hard spheres differed in weight (hard: 4.8 g, soft: 0.71 g), participants might also have used the total weight of the bundle as a cue to perform experiment 1b, especially when the target was a hard, thus heavy, sphere. Because the spheres lay on a display in Experiment 2, no weight cues were available. The same participants as in Experiment 1 took part and the order of Experiment 1 and Experiment 2 was counterbalanced between participants.

### Methods

#### Apparatus

**Figure 5:**
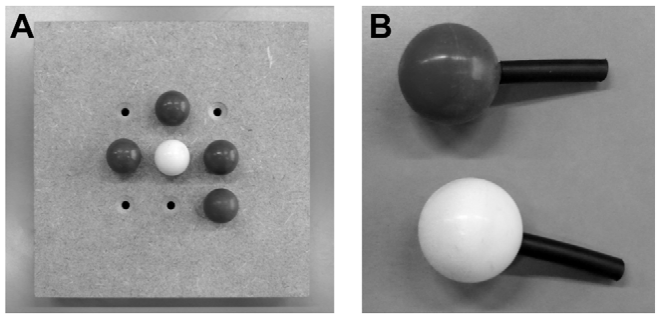
Experimental set-up of **Experiment 2**. A. Stimulus display with 4 hard distractors and one soft target. B. A hard and a soft sphere on poles.

A 3×3 grid was used to present the stimuli (see Fig. 5). In each of the resulting 9 positions, a sphere could be placed. The filled grid was 6.8 cm by 6.8 cm, so it would fit the size of the hand. A small rubber pole was attached to the spheres, so they could fit into small holes that had been drilled in the centre of each position. This ensured that the spheres remained in place, but still could be compressed. The items used were the same hard and soft (hollow) spheres as in experiment 1b. On the display, 3, 5, 7 or 9 spaces were filled with items, leaving the other positions empty.

To measure the reaction time, the grid was placed on the weighing scale. As the participants touched the grid, the weight change started the clock. The end of the reaction time was determined by a vocal response, recorded with a headset, similar to Experiment 1.

#### Task and Procedure

Two conditions were performed in Experiment 2: in one case, blindfolded participants had to determine the presence of a hard target among soft distractors, whereas in the soft-target condition they had to search for a soft target among hard distractors. In each condition, 20 trials per number of items (3, 5, 7 or 9) were performed, of which half of the trials contained a target. The number of items and the target-present and target-absent trials were randomized in a condition. The locations of the target and distractors were also random, but for the target-present trials each configuration was unique. However, since there were more trials (10) than possible configurations (9) in the target-present trials with 9 items, each configuration was used once and a 10th configuration was chosen randomly. In the case of 9 items with no target present, of course only a single configuration was possible. The order of target identity was randomized between participants in a way that an experiment was started with the hard- or soft-target condition equally often.

Participants were instructed to initially put down their whole hand flat upon the grid, but if necessary they could then lift their hand and press again or use their fingers to individually touch the items. They were told to only press down on the spheres or press from the sides. Between trials they held their hand on a resting platform. Before a condition, participants performed a practice session with the same requirements as in Experiment 1. The maximum number of practice trials that were necessary was 33 trials. Each condition was performed on a separate day.

#### Analysis

One trial (0.06%) was removed from the analysis due to a measurement error. Outliers were removed from further analysis using the same criterion as in Experiment 1. Next, a straight line was fitted through the reaction time data as a function of number of items for each participant. The slope and the intercept of the regression were calculated for the type of target and target-present and target-absent trials separately.

The position of the distractors could influence the ability to detect the target, especially in the case of a soft target. To investigate this, the relation between the number of adjacent distractors and the reaction time was calculated. A regression line was fitted through the averaged reaction time against the number of distractors that directly neighboured the target (horizontally or vertically; direct distractors). A similar fit was made for all adjacent distractors, that is, including the ones that diagonally enclosed the target (+diagonal distractors). A weighted fit was used to adjust for the difference in number of trials for each number of adjacent distractors. In addition, the relation between the reaction time and the location of the target was determined by averaging the reaction time for each of the 9 target locations.

A 2 (target type)×2 (target presence) repeated measures ANOVA was performed on the search slopes and intercepts. Furthermore, a 2 (target type)×2 (direct/+diagonal distractors) repeated measures ANOVA was conducted on the slopes and intercepts of the reaction time against the number of adjacent distractors. Finally, a 2 (target type)×3 (horizontal position)×3 (vertical position) repeated measures ANOVA was performed on the reaction times averaged over target location. The significance value was set at 0.05. If the sphericity assumption was violated according to Mauchly's test, a Greenhouse-Geisser correction was used. Post-hoc tests were performed using paired-sample *t*-tests with a Bonferroni correction. Only relevant comparisons were made.

### Results

#### Errors

As in Experiment 1, participants more often made errors in target-present trials (false negative errors) than in target-absent trials (false positive errors). This indicated that participants more often missed a target, than imagined a target that was not there. Furthermore, far more errors were made in the search for a soft target than in the search for a hard target (see Table 3).

**Table 3:** Percentage of errors made in Experiment 2, for each number of items separately.

		3	5	7	9
hard	Present	2	0	0	3
	Absent	0	0	1	0
soft	Present	1	1	10	8
	Absent	0	0	0	1

#### Reaction times

**Figure 6:**
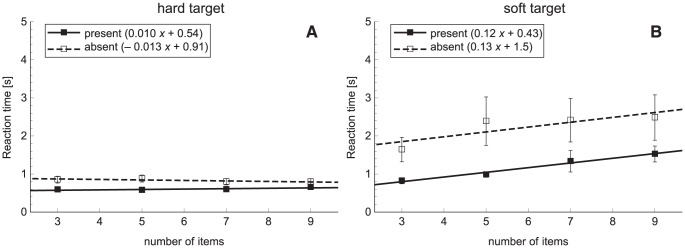
Search slopes for **Experiment 2** **for a hard target (A) and soft target (B), for target-present and target-absent trials separately.** Error bars represent standard errors.

Search slopes of Experiment 2 are displayed in Fig. 6. The values of the slopes and intercepts can also be found in Table 2. An ANOVA on the slopes demonstrated an effect of target type (*F*_1,9_ = 10.1, *p* = 0.011). Steeper slopes were seen in the search for a soft target compared to a hard target. Only the slope for target-present trials in the soft-target condition was significantly different from zero.

In the analysis of the intercept, effects of target type (*F*_1,9_ = 5.2, *p* = 0.048) and target presence (*F*_1,9_ = 11.6, *p* = 0.008) were found. The intercept was lower in the search for a hard target than in the search for a soft target. In addition, the intercept was higher when the target was absent than when it was present.

**Figure 7:**
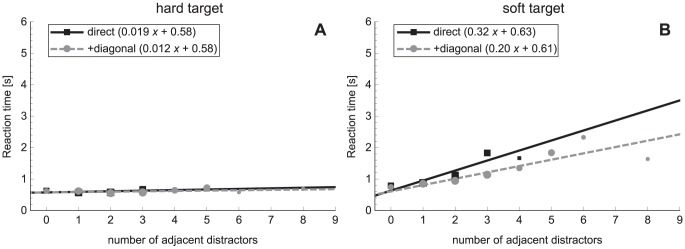
Relation between reaction time and number of adjacent distractors for a hard target (A) and a soft target (B). Lines are plotted separately for direct distractors (black) and direct+diagonal distractors (grey). Point size indicates the weight of the point (number of trials) according to a logarithmic scale.

Based on the results of Experiment 1b, it was not expected to find a difference between the search for a hard and a soft target, i.e. a search asymmetry. However, the differences found might be explained by the location of the target on the display. When hard distractors surround a soft target, participants are impaired to compress the target, since their hand is blocked by the distractors. To investigate this, the relation between the reaction time and the number of adjacent distractors was calculated. The regression lines are plotted in Fig. 7. Slope values and intercepts are displayed in Table 4. It can be clearly seen that the reaction time does not depend on the distractor position in the search for a hard target. When the soft sphere is the target, the reaction time increases with the number of adjacent distractors. This is confirmed by a regression analysis. The slopes in the hard-target condition were not significantly different from zero, whereas both the slopes for direct and for +diagonal distractors were significant in the search for a soft target. An ANOVA on the slopes produced main effects of target type (*F*_1,9_ = 7.8, *p* = 0.021) and distractor position (*F*_1,9_ = 18.3, *p* = 0.002). Shallower slopes were found in the search for a hard target compared to the search for a soft target. However, in the post-hoc tests of the interaction of target type × distractor position (*F*_1,9_ = 17.7, *p* = 0.002), no significant differences between the target types were found (direct: *p* = 0.059, +diagonal: *p* = 0.144). The slope of the direct adjacent distractors was steeper than the slope including the diagonal distractors, but only when a soft target was to be searched for (*p* = 0.008). No significant effects were found in the analysis of the intercept.

**Table 4:** Slopes and intercepts for the regression of reaction time against the number of adjacent distractors.

	Slope (s)		Intercept (s)	
	direct	+diagonal	direct	+diagonal
hard	0.019	0.012	0.58**	0.58**
soft	0.32*	0.20**	0.63*	0.61**

* p<0.05,

** p<0.01.

**Figure 8:**
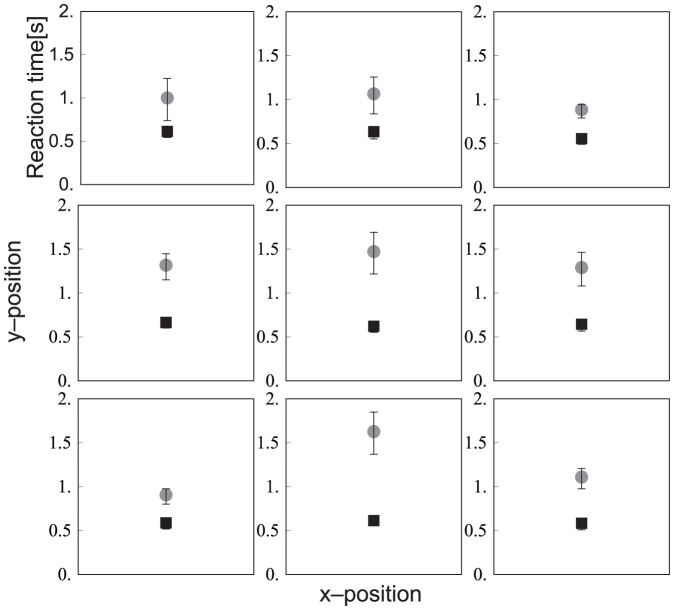
The reaction time plotted at each possible target location, for hard (black square) and soft (grey disc) targets separately. Each plot is located at the corresponding x- and y- position of the display. Error bars represent standard errors.

Besides the position of the distractors, also the location of the target might have been of influence on the reaction time, as illustrated in Fig. 8. Therefore, a 2 (target type)×3 (vertical position)×3 (horizontal position) ANOVA was conducted on the reaction times for target-present trials. As found above, the reaction time was higher in the search for a soft target (effect of target type; *F*_1,9_ = 23.6, *p* = 0.001). An interaction between target type × vertical position (*F*_2,18_ = 6.6, *p* = 0.007) revealed that the difference between the search for a hard target and a soft target was not seen for the upper row, only for the middle and lower rows (*p* = 0.198, *p* = 0.015, *p*<0.001 respectively). An effect of vertical position (*F*_1.3,11.6_ = 9.3, *p* = 0.007) demonstrated that the reaction time was higher if the target was in the middle row, than if it was in the upper row. The interaction of target type × vertical position showed that this was only the case when a soft target was searched for (*p*<0.001). Also, an interaction of vertical position × horizontal position (*F*_4,36_ = 3.0, *p* = 0.033) was found. Post-hoc tests indicated that there was a difference between a target on the upper row and the lower row, when it was located in the middle column (*p* = 0.005).

## Control Experiment

There existed a very subtle difference in texture between the hard and the (middle-) soft spheres, where the hard spheres were somewhat smoother. To investigate whether the texture difference could have explained the results, a control experiment was performed in which participants had to quickly classify the stimuli.

### Methods

#### Participants

Ten new right-handed participants (5 males) with a mean age of 24±3 years took part in the experiment. They had no previous experience with the stimuli. All signed an informed consent form and were paid for their contribution.

#### Apparatus

The same experimental set-up as in Experiment 2 was used. Only the hard and soft spheres were used.

#### Task and procedure

One item was placed on the display and participants had to classify the item in two separate tasks. In the compliance task, they had to say whether the item was hard or soft and in the texture task, participants had to choose between smooth or rough. The order of the tasks was counterbalanced between participants. Participants were instructed, similar to Experiment 2, that they should initially press their hand on the stimulus, but could then also use their fingers. They should answer to which category (e.g. hard or soft in the compliance task) the stimulus belonged, and do this as quickly as possible, but also with as few mistakes as possible. The reaction time was recorded, using the weighing scale and the head-set. Participants performed 10 trials in each task, with 5 trials for each item type. Incorrect answers were repeated. Before the start of the task, the items were pressed or stroked once or twice by the experimenter against the hand palm in order to indicate which item belonged to which category.

## Results and Discussion

A 2 (task)×2 (type) repeated measures ANOVA was performed on the reaction times. An effect of task (*F*_1,9_ = 12, *p* = 0.007) revealed larger reaction times in the texture task than the compliance task (texture: 1.4±0.18 s, compliance: 0.68±0.029 s). Also, more mistakes were made in the texture task (texture: 9%, compliance 0%). This shows that texture discrimination was more difficult than compliance discrimination, making it unlikely that participants used texture as a cue to perform the search task. In addition, participants reported that they found it very difficult to distinguish the items based on texture. Some even used the correlation with compliance to perform the task, once they discovered it, because they could not feel the texture difference. No effect of type was found.

### General Discussion

The aim of this study was to investigate the saliency of hardness and softness in an active haptic search task. In Experiment 1, a grasping task was performed in which participants had to grasp a bundle of spheres and determine whether a target was present. The target could be a hard sphere among soft distractors, or a soft sphere among hard distractors. The difference in compliance between target and distractors could be small (experiment 1a) or large (experiment 1b). In short, the search was very efficient when the difference between the target and distractor was large, but inefficient when the difference was small. This result was found both for hard and for soft targets. In the following paragraphs these results will be further elucidated.

In Experiment 1, there were no differences in the search for a hard or soft target. This means that there is no search asymmetry between the two search tasks. Therefore, it is not easier to search for a hard target than for a soft target or vice versa. These results are in agreement with the study of Lederman and Klatzky [5], who also did not find a search asymmetry.

When a hard target was searched for among soft distractors, reaction times were short. In addition, the slope of the reaction time against the number of items (search slope) that were searched was shallow. This means that the time it takes to determine the presence of a target does not depend on the number of items in the hand. A completely different behaviour was seen when the distractors were middle-soft. The reaction times were much higher and the search slope was almost half a second per item in target-present trials, or a second per item in target-absent trials. This indicates that the items had to be explored one by one, thus in a serial way, which would explain the increase in reaction time with more distractors. The behaviour of participants in Experiment 1 confirmed this explanation. If the difference between target and distractors was small, participants often dropped spheres out of their hand, whereas this rarely happened when the difference was large.

The same results were found in the search for a soft target. If a soft target was searched for among hard distractors, the search slope was flat and the reaction times were low. This implies that the reaction time is independent of the number of items. This was not the case in the search for a middle-soft target among hard distractors. The reaction time increased markedly with more items, especially when no target was present. In addition, participants more often dropped one or more spheres out of their hand when searching for a middle-soft target. This indicates that the items were searched for with a serial strategy.

Taken together, the results show that hardness and softness can be efficiently searched for when the difference between target and distractors is large. However, the slope values of experiment 1b (hard: 80 ms/item, soft: 48 ms/item) are somewhat high compared to previous studies that investigated the saliency of haptic properties in an active search task, like roughness (20 ms/item; [11]), 3-D shape (25 ms/item; [13]), temperature (32 ms/item; [12]) and movability (39 ms/item, [14]). Still, the slopes in experiment 1b were very low compared to experiment 1a that clearly showed a serial search. From this we conclude that hardness and softness can be efficiently searched for and that they are salient features.

In theory, one would expect a 2∶1 relationship in the search slopes for target-absent and target-present trials if the search strategy were serial. If the items are explored one by one and the search is stopped when a target is found, then on average half of the items are explored in target-present trials, whereas all items need to be searched in target-absent trials. So, search slopes in target-absent trials would be twice as high as in target-present trials. In experiment 1a, the ratios are on average close to 2∶1, which would indicate a serial search. On the other hand, for experiment 1b the ratios are much higher. Although it must be kept in mind that there was no significant difference between target-present and target-absent slopes, the target-absent slopes seemed to be much higher in this experiment. This might be explained by participants searching longer when the target is absent to make sure they did not miss it. Especially with a large number of items, the target could easily have been hidden between the distractors. This raises the question about the influence of location on the reaction times; it might have been more difficult to detect a target in the middle of the bundle. Still, the target was located randomly and at various positions in the bundle and participants were able to easily rearrange the spheres in their hand, so this cannot account for the differences found between the conditions.

Another point of discussion is that participants might not have based their judgement on compliance alone. Although we tried to avoid other perceptual differences between the item types besides compliance, possible other cues, like weight or texture, should be considered. First, there were some small differences in texture between the item types, where the hard spheres were somewhat smoother. Therefore, a control experiment was performed, which showed that participants classified the stimuli much faster and with fewer errors based on compliance than on texture. The difference in texture was much harder to perceive, making it unlikely that participants based their judgement on texture. Furthermore, the soft and middle-soft spheres were made of the same rubber material, giving the same texture difference with the hard spheres. If participants had performed the task using texture as a useful cue, one would also expect low search slopes in experiment 1a, which was not the case. So, it does not seem that texture played a role in the search tasks.

Secondly, the soft spheres were hollow, whereas the hard spheres were solid. It cannot be ruled out that ‘hollowness’ was perceived instead of softness. With our current stimulus materials it was not possible to make soft items that were also solid, but it could have influenced the results. Nevertheless, it might be questioned what hollowness is, whether it can be perceived and how it differs from the perception of softness.

A third possible factor that could have influenced the perception of hardness in Experiment 1 was the weight difference between the hard and soft items. There was a small weight difference between the hard and middle-soft items, but the difference between a bundle with three items (lowest number of items possible) with and without a target was less than a gram, or about 6%. This is around the Weber fraction for weight discrimination (3–12%, [18]), and thus near the JND. Therefore, weight was probably not used to determine the presence of a target in experiment 1a.

In experiment 1b, with hard and soft targets, the weight difference was about 4 g, which is a very distinctive difference and could reveal the presence of the target very easily without the need to determine the compliance of the spheres. This probably did not play a large role in the search for a soft target, because the target weight is small compared to the distractors and participants did not know the number of items presented. A lighter weight could indicate a target, but also a small number of items. It might have only played a role with three items, because this number can be subitized [19], which means that the number of items in the hand can be determined instantaneously and without counting. For higher numbers, the items would have to be counted, which would take more time than the short reaction times found in this study. Thus, weight was not a reliable cue to be used by participants in the soft-target condition of experiment 1b and could therefore not explain the pop-out effect.

However, in the search for a hard target, the presence of the target causes a large increase in total weight, which could have been used as a cue. On the other hand, if weight had popped out instead of compliance, one would expect a lower search slope in the search for a heavy item among light distractors than the other way round. Yet, the slope in the search for the hard, heavy target was higher than in the soft, thus light, target search. To exclude the possible influence of this weight difference, the spheres were placed on a display in Experiment 2. Participants had to press their hand on the spheres to determine whether a target was present. When a hard sphere was the target, the search was very efficient. The search slopes were flat and reaction times were low. The search slope in this condition appeared to be even smaller than when the hard target was searched for by grasping. This indicates that the pop-out of a hard target in Experiment 1 was not caused by the use of weight differences as a cue.

The difference in search efficiency between the search for a hard target in Experiment 1 and Experiment 2 might be explained by the positioning of the items in each task. When grasping a bundle of items, the hard target may be hidden in the centre of the bundle, so the target is not pressed directly. In the task in Experiment 2, the items are neatly arranged and it is possible to press all the items at once and quickly locate the hard target.

The search slope for the hard-target condition in Experiment 2 is comparable to the search slopes Lederman and Klatzky found in a task where compliant objects were pressed against the fingertips [5]. They found a slope of 3 ms/item in the search for a hard target and 10 ms/item in the search for a soft target. Thus, our results show that the pop-out of hardness and softness cannot only be found in passive touch, but also in active, haptic perception.

Unexpectedly, we found a search asymmetry in Experiment 2. Searching for a soft target was more difficult than finding a hard target and high search slopes were found. This could not be explained by a more frequent exposure to hard spheres, since all participants showed this asymmetry, regardless of the order in which they did the experiments. The search slopes in the search for a soft target in this experiment were much higher than the slopes in the grasping task. However, this does not necessarily mean that softness did not pop out in the second experiment. It is more likely that the location of the target played a large role in the detection of the target. There was a large dependence of the reaction time on the number of adjacent distractors in the search for a soft target, whereas there was no relation when a hard item was the target. It seemed that directly adjacent distractors had a larger influence than diagonal distractors, because the slope of the reaction time against the number of adjacent distractors was steeper if only directly adjacent distractors were counted.

Furthermore, if the target was located close to the fingers, the target was found quite fast, whereas it was more difficult when the target was located near the palm of the hand. When the target is located near the palm of the hand and/or many hard distractors surround it, the hand is blocked by the distractor items. The target cannot be compressed enough to perceive it as a soft target. Since there were also empty spots on the display, participants might have mistaken the target for an empty space. They had to press again with their fingers to make sure no target was there and thereby increased their reaction time. So, the higher reaction times in the search for a soft target were not caused by the inability to locate the target, but by the inability to compress it.

An interesting finding is that the slopes of target-absent and target-present trials did not differ in Experiment 2 for the soft-target condition. Like explained above, in a serial search one would expect to find a higher slope in the target-absent trials, because the search stops when a target is found. This was seen in Experiment 1, but not in Experiment 2. In the search for a hard target, it was so obvious when a target was present that one could be very sure there was no target when it was not immediately felt. In the soft-target condition, the reaction times are much higher for target-absent trials than target-present trials, but by a constant value, i.e. a higher intercept in the search slope. Possibly, participants did not always search item by item, but used a row-by-row strategy. In a pure row-by-row strategy, the slope will not be higher in target-absent trials, because the same number of rows need to be searched for each item number. In target-absent trials, participants would have to search all rows, but in target-present trials they can stop once a target is located in the upper row(s), leading on average to a lower intercept. This is confirmed by the finding that lower reaction times were found if the target was located in the upper row. In Fig. 8, an exception can be seen in the lower left corner, where reaction times were quite low for the soft-target condition. At this position, the thumb was located, which participants often pressed against the side of the item to determine whether a target was present. Another factor that could have caused the large increase in reaction time in target-absent trials, even with few items, is that participants might have been unsure whether they felt all the items and perhaps misplaced their hand on the display.

To summarize, a pop-out effect for both hardness and softness was found. When the items are arranged in a 2D-display the search is even easier for a hard target. Because weight cues are not possible in this set-up, the pop-out effect was not caused by the weight differences in the items. If items were placed on a 2D-display, the search is more difficult for a soft target, but this is caused by the inability to compress the target, since the hand is blocked by the hard distractors. In conclusion, both hardness and softness are salient haptic features. This holds for passive and active search and for different exploratory procedures that are used for compliance perception. This knowledge could be useful for the development of simulation systems. These tools can, for example, be used for the training of palpation in medical students.
